# A novel ISCA2 variant responsible for an early-onset neurodegenerative mitochondrial disorder: a case report of multiple mitochondrial dysfunctions syndrome 4

**DOI:** 10.1186/s12883-019-1387-2

**Published:** 2019-07-06

**Authors:** Milad Eidi, Masoud Garshasbi

**Affiliations:** Department of Medical Genetics, Faculty of Medical Sciences, Tarbiat Modares University, Tehran, Iran

**Keywords:** ISCA2, Iron-sulfur clusters, Fe-S clusters, Energy production, Leukoencephalopathy, MMDS4, Glycine encephalopathy, WES

## Abstract

**Background:**

Multiple Mitochondrial Dysfunctions Syndrome 4 (MMDS4) is manifested as a result of *ISCA2* mutations. ISCA2 is a vital component of 4Fe-4S clusters assembly machine. Therefore, in MMDS4 patients, deficient mitochondrial respiratory chain complexes I and II, Aconitase and Succinate dehydrogenase of Kerbs cycle and Lipoic Acid Synthetase in the biosynthesis of lipoic acid are expected.

**Case presentations:**

A 7 months boy in an Iranian consanguineous family with progressive neurodegenerative problems was referred to us. Primarily, general laboratory tests, Abdomen ultrasonography and brain magnetic resonance imaging were performed. In order to find out the genetic problem in this case Whole Exome Sequencing (WES) following by Sanger sequencing was carried out. A novel variant (c.355G > A, p.Ala119Thr) in *ISCA2* gene was identified by WES in the proband. Confirmation and segregation in the family for this variant was performed by Sanger sequencing. In-Silico prediction of the ISCA2 secondary structure showed that a helix motif in the Fe-S biosynthesis domain of ISCA2 protein will be eliminated as a result of this variant.

**Conclusions:**

We reported the first patient with *ISCA2* variant in Iranian population and the third one in the world reported for *ISCA2* gene, so far associated with early-onset mitochondrial neurodegeneration. However further functional studies on this variant or finding it in other patients with similar clinical problems are needed to confirm the pathogenicity of this variant.

**Electronic supplementary material:**

The online version of this article (10.1186/s12883-019-1387-2) contains supplementary material, which is available to authorized users.

## Background

Mitochondria contain about 1500 functional proteins but only 13 of them are coded by the mitochondrial genome (mtDNA), and the rest are coded by the nuclear genome (nDNA). This fact justifies the inheritance patterns of mitochondrial diseases [1, 2]. mtDNA mutations are responsible for approximately 80% of adult mitochondrial diseases and only 20–25% of childhood cases. Accordingly, most cases of childhood mitochondrial diseases are due to mutations in nDNA [3]. Mitochondrial Iron-Sulfur Clusters (ISCs) are the necessary cofactors for pivotal life processes, such as electron transport in energy production as their main role, gene expression regulation, DNA maintenance, sulfur delivery in the process of lipoic acid synthesis, and the antiviral responses [4].

The production of ISCs in the mitochondria requires lots of proteins in stages like assembly, maturation and delivery to the targets. Defects in any of these proteins in this pathway play a major role in reducing the de novo production of ISCs. Up to now, Studies in yeast and human cell lines have shown the function of at least 17 protein components in the mitochondrial ISC assembly system, which mutation in any of them can cause severe disorders in humans [5–9].

A class of mitochondrial diseases that defect energy production and also affect the white matter of brain are MMDSs (Multiple Mitochondrial Dysfunctions Syndrome), of which four types have been introduced. *NFU1* (MIM #608100) [10, 11], *BOLA3* (MIM #613183) [10, 12], *IBA57* (MIM #615316) [13] and *ISCA2* (MIM #615317) [9] genes are responsible for 4 types of MMDSs, respectively. Recently, it has been argued whether *ISCA1* mutations could be considered as the cause of another type of MMDSs or not [14, 15]. Common features of all of these dysfunctions are neurodevelopmental delay, defected lipoic acid biosynthesis, seizures, lactic acidosis, weakness, leukodystrophy, hypotonia, dystonia, and autosomal recessive mode of inheritance [6, 9, 10, 12–15].

In this report, we present a novel variant in the *ISCA2* gene which is the first variant in this gene will be reported in Iranian population and the third variant in the world reported for *ISCA2* gene so far.

## Case presentation

**Fig. 1:**
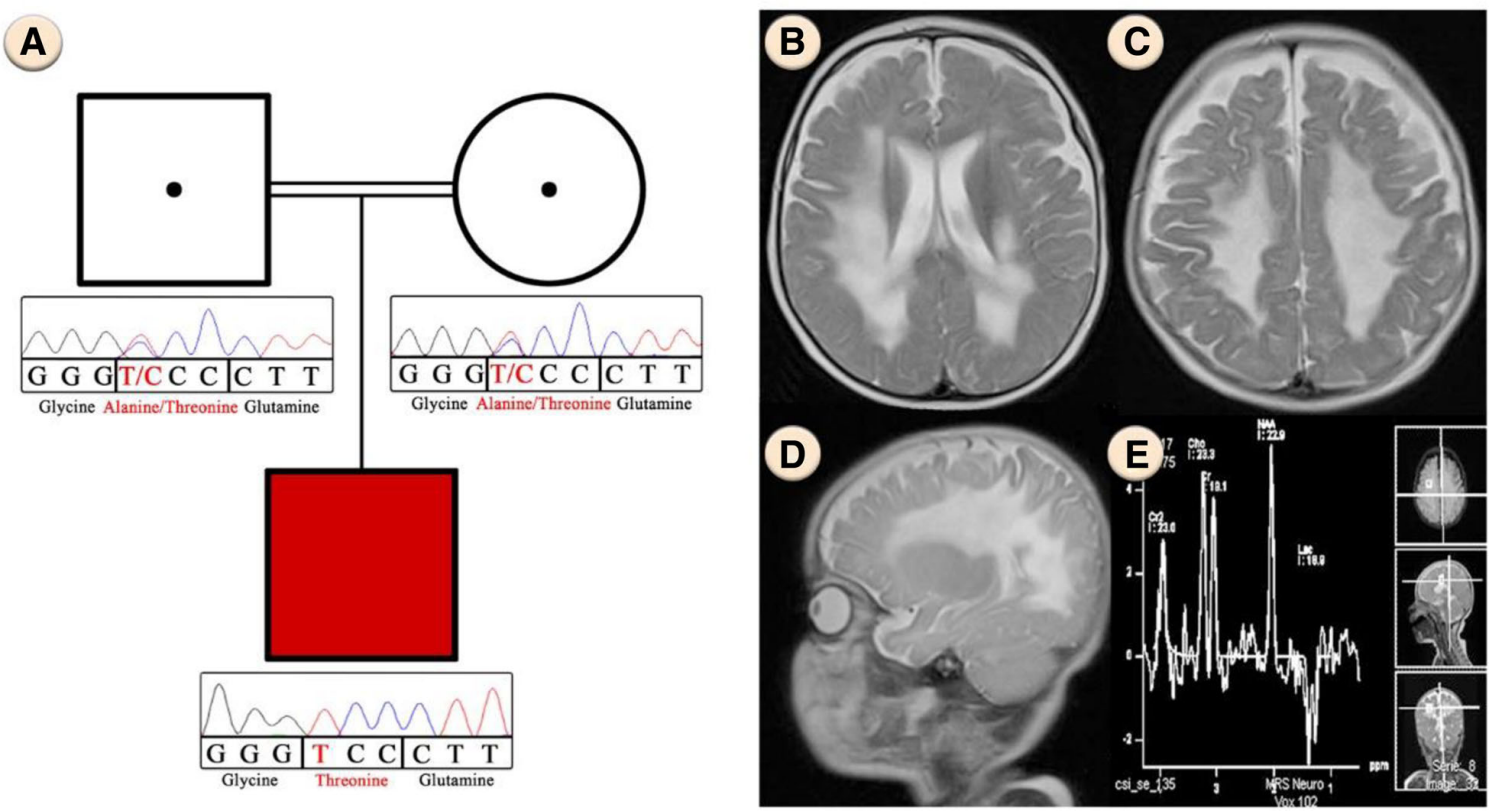
**a** Pedigree of the family. Chromatograms represent homozygous and heterozygous state of c.355G > A variant in *ISCA2* in the patient and his parents respectively. **b**, **c** Nearly symmetrical involvement of white matter in the axial view **d** Sagittal T2 view that shows extensive white matter signal change **e** Magnetic Resonance Spectroscopy that shows an increase in lactate levels and a relative increase in the choline with respect to N-acetyl aspartate (NAA)

The proband (Fig. 1a), a boy who is the first child of an Iranian consanguineous family, was born after an uneventful pregnancy, but seven month after birth he developed some problems such as, malaise, insomnia, irritability, muscle stiffness and hypotonia. All general tests of the patient, including Complete Blood Count, Calcium, Phosphorous, Alkaline phosphatase, Total Vitamin D and Urine Analysis were normal. The patient’s abdomen sonography showed a normal size of kidney and spleen for his age. Brain magnetic resonance imaging showed nearly symmetrical abnormal changes in the areas of the periventricular white matter that was distributed in both centrum semiovale and has involvement of both middle cerebellar peduncles (Fig. 1b, c, d). Additionally, magnetic resonance spectroscopy which measures the amount of brain metabolites, indicated an increase in the lactate levels and a relative increase in the choline with respect to N-acetyl aspartate (NAA) in the periventricular regions of the patient’s brain, which is mostly reported in mitochondrial diseases (Fig. 1e).

### Sample collection and DNA extraction

DNA was extracted from the peripheral blood of the patient, his parents, and one of his aunts using the Roche DNA Extraction Kit (Product No. 11814770001). The quality and quantity of the extracted DNAs were examined by Nanodrop and running on the gel.

### Whole exome sequencing

Whole Exome Sequencing (WES) analysis was performed for the proband, where approximately 37 Mb (214,405 exons) of the Consensus Coding Sequences were enriched from fragmented genomic DNA by > 340,000 probes designed against the human genome (Nextera Rapid Capture Exome). The generated library was sequenced on the Illumina Hiseq2500 platform to an average coverage depth of 175X. All in all, 95.9, 98.2 and 99.8% of exons were covered with at least 20, 10 and one reads respectively (Additional file 1: Table S1).

### In-silico bioinformatics analysis

An end to end in-house bioinformatics pipeline including base calling, adapters trimming, FASTQ file quality controls, primary filtering of low quality reads and probable artefacts were applied. Subsequently, reads alignment to reference human genome (hg 19), variant calling, recalibration of quality scores, annotation and filtration of variants were performed by HISAT2 [16], Genome Analysis Toolkit (GATK) [17], Annovar tool [18] and MySQL 8.0, respectively. It should be noted that dbSNP (build 150), 1000 Genome Project (1000 GP) and Exome Aggregation Consortium (ExAC) [19] databases were used for filtering purposes. The clinical significances of variants were taken from ClinVar (September 5, 2017), list of patient’s phenotype-related genes was also extracted from CentoMD database [20]. The probable effects of prioritized variants were predicted using PolyPhen-2 [21], SIFT [22], PANTHER [23], Mutation Taster [24], PMut [25], Human Splicing Finder, SNAP [26] and PROVEAN [27] algorithms. Finally, filtering of Iranian population variants were performed using Iranome database (http://www.iranome.ir). Protein residue conservations were also examined using the ConSurf server (http://consurf.tau.ac.il/2016/) [28]. Secondary structures of Wild-type and Mutant *ISCA2* proteins were predicted by GORIV [29].

### Sanger sequencing

Sanger sequencing was used to validate the identified variant in *ISCA2* in the patient, his parents, and one of his aunts (Applied Biosystems). Primer sequences and PCR conditions are available upon request.

## Results

After quality control and alignment, single nucleotide variants and small indel variants were called by GATK (74353 variants). Subsequently, GATK recommended filters for quality score recalibrations were applied and rest of the variants were annotated by Annovar tool (67205 variants). Because only one affected existed in this consanguineous family, both of heterozygous (42628 variants) and homozygous (24577 variants) variants, was considered.

**Fig. 2:**
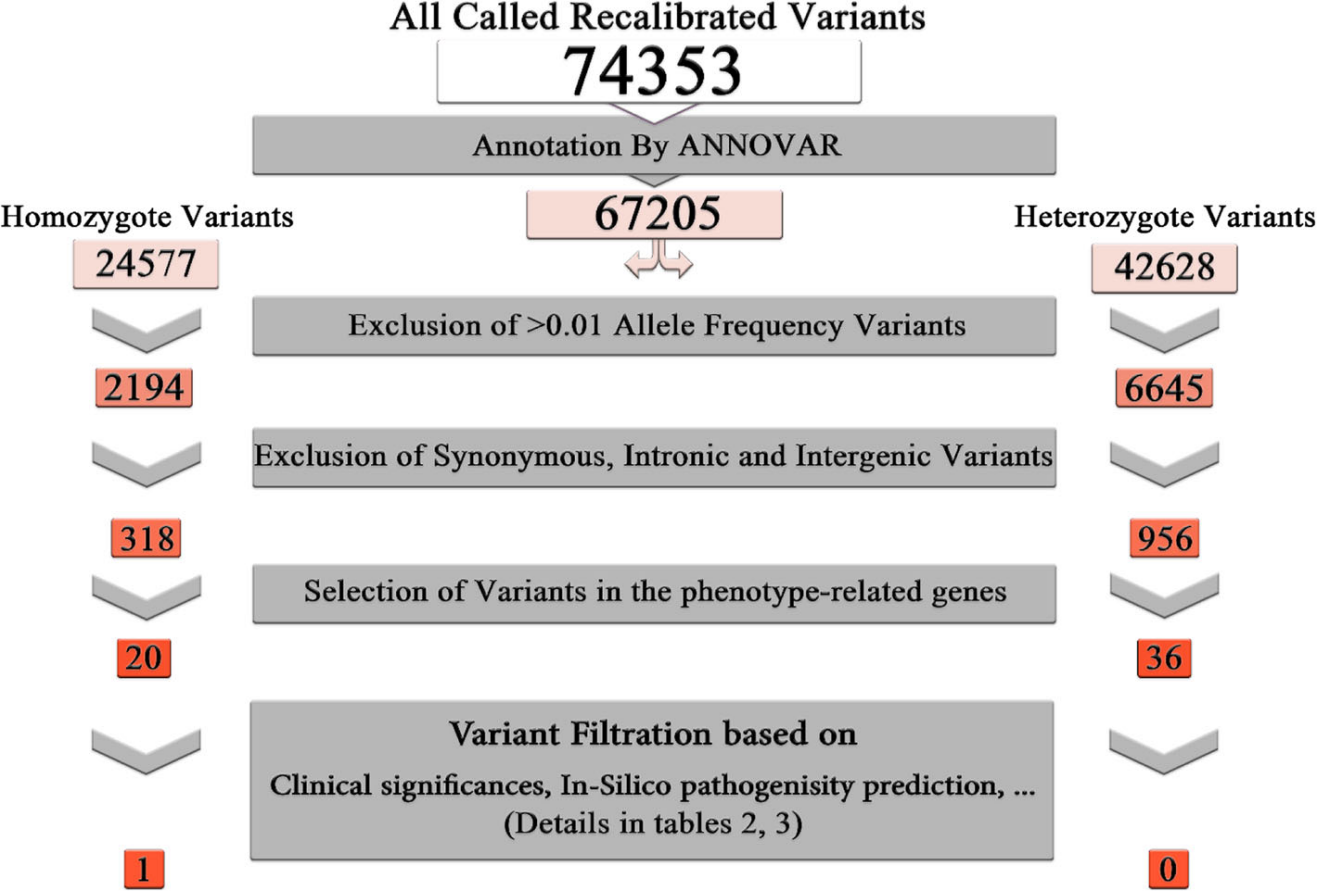
Flowchart of variants filtration process

In the filtration process, by excluding the variants with allele frequency greater than 1% in 1000GP, ExAC, dbSNP databases only 2194 homozygous and 6645 heterozygous variants remained. Then, variants that located in the exonic and splicing regions were chosen and Synonymous variants were excluded which ended up with 318 homozygous and 956 heterozygous variants. After that, we chose only variants located in the patient’s phenotype-related genes (20 homozygous and 36 heterozygous variants). The details of this filtration steps are presented in Fig. 2.

Finally heterozygous variants were prioritized according to the expected inheritance mode of mutated genes, clinical significances and in-silico effect predictions (Additional file 1: Table S2 and Table S3). In other word, heterozygous variants identified in the genes which only homozygous variants with pathogenic effect for it have been reported in the literature, have not been considered. Otherwise, heterozygous variants were judged based on clinical significances and in-silico effect predictions, respectively. The homozygous variants were filtered only based on clinical significances and in-silico predictions. Also some variants were ruled out based on ethnic matched allele frequency database.

**Fig. 3:**
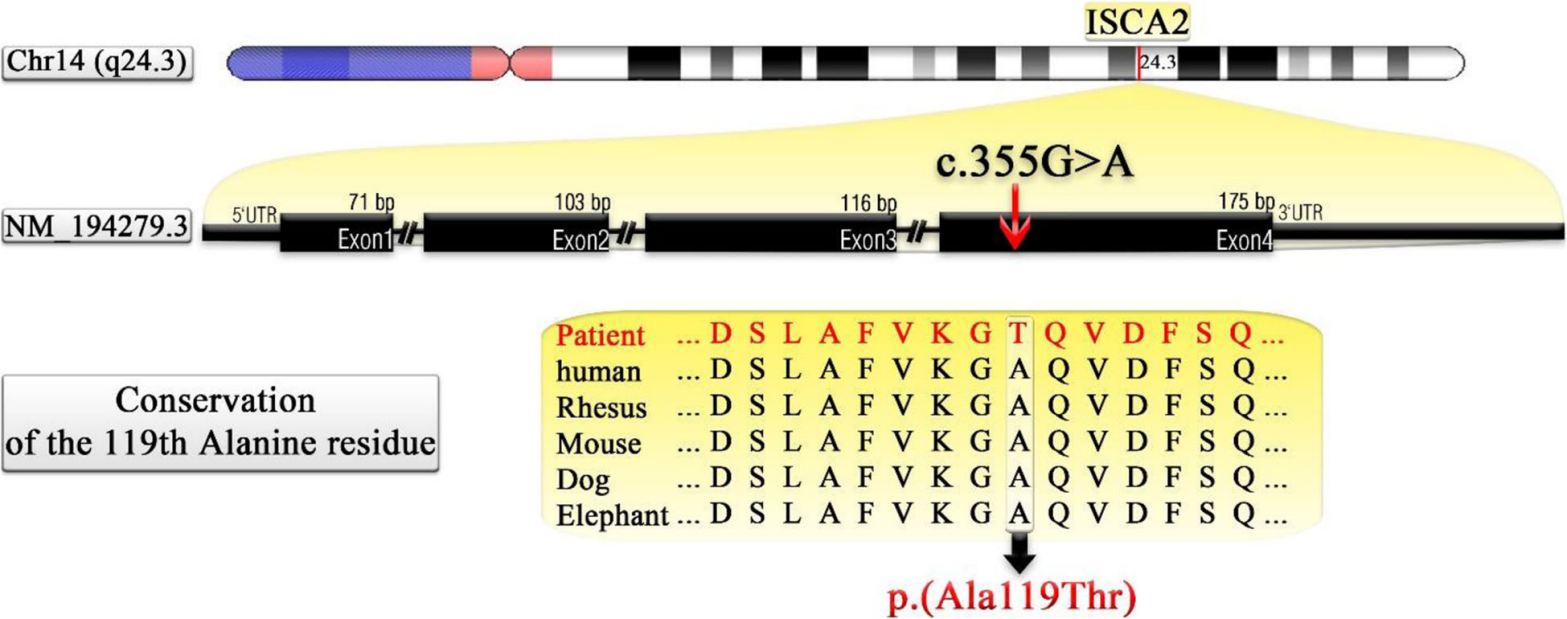
Gene structure of *ISCA2* and position of the identified pathogenic variant. (Introns are not drawn in scale)

We concluded that the homozygous variant [c.355G > A, p.Ala119Thr] in *ISCA2* gene (NM_194279.3), which affects a highly conserved domain of *ISCA2* (encoded by exon 4) leads to the severe phenotypes observed in the patient (Fig. 3). The PROVEAN algorithm predicted that this variant is Neutral but other algorithms such as Mutation Taster, PANTHER, PolyPhen-2, SIFT, SNAP, PMut suggested that it is, in fact, detrimental to protein function. Sanger Sequencing results showed co-segregation of the c.355G > A variant with the disease in the family. Altogether these evidences strongly suggest the causality of this variant in the pathogenicity of infantile leukoencephalopathy in this family.

## Discussion and conclusions

Each person has ~ 400–500 protein-modifying variants in the coding regions of his or her genome which makes it highly challenging in diagnostic to choose the pathogenic culprit variants among the others. In the current report, due to lack of functional analysis, we cannot be completely sure about the diagnosis, but the evidences provided here highly suggest that the c.355G > A variant in *ISCA2* is a causative variant in this family.

The [4Fe-4S] clusters are prosthetic groups of a wide spectrum of proteins, such as Mitochondrial Respiratory Chain (MRC) complexes I and II, Aconitase and Succinate dehydrogenase of Kerbs cycle and Lipoic Acid Synthetase in the biosynthesis of lipoic acid. Also, lipoic acid is a cofactor of 5 multimeric enzymes, which are taking part in energy metabolism and catabolism of Lysine and Glycine, generally [30, 31]. Considering the cascading events, it can be inferred that deficiency in the *ISCA2* disrupts the energy metabolism and Glycine catabolism. Hyperglycinaemia causes Glycine encephalopathy, which is one of the fatal features of patients with defected *ISCA2*.

Furthermore, a functional study in HeLa cells have stated that in the *ISCA2* depleted cells by RNA interfering technology, the function of [4Fe-4S] clusters-free proteins may also be affected, like MRC complex IV, which is probably due to a pleiotropic effect of deficiency in assembling of MRC complexes I and II and their supercomplexes [32]. An example of this pleiotropic effect has been reported in an *ISCA2* mutated patient [33].

Al-Hassnan et al. for the first time reported a d.G74961032A; c.G229A; p.G77S mutation in *ISCA2* gene in 6 patients of 5 consanguineous families with following phenotypes: failure to thrive, spasticity, optic atrophy, severe leukodystrophy, and neurological regression. The age of onset in these patients was 3–7 months and the maximum lifespan was 5 years. The uniformity of mutation in all the families in their report indicated the existence of a founder effect. Later Al-Fadhel et al., in a retrospective review, reported 10 additional patients with the same mutation in Saudi Arabia [34].

The second mutation in the *ISCA2* has been reported by Toldo et al. in an Italian family, recently. The case was a girl with compound heterozygote state for a nonsense and a missense mutation in *ISCA2* gene. Interestingly, their biochemical analyses showed that the [4Fe-4S] clusters-free MRC complex IV function has also been affected [33]. Here we report the third mutation in the *ISCA2* gene.

**Fig. 4:**
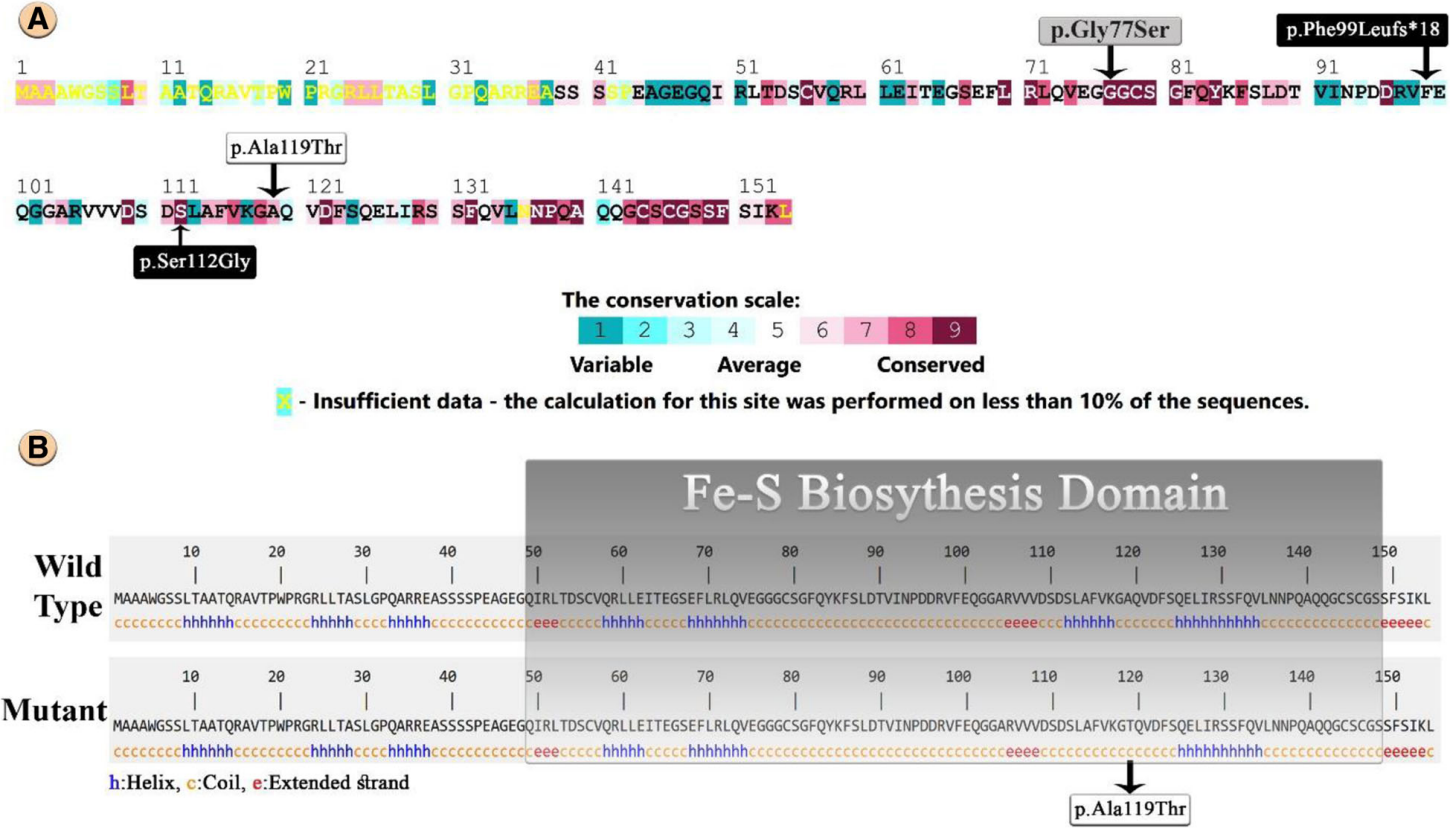
**a** The amino acid residues of ISCA2 colored based on conservation scores produced by ConSurf database and presentation of all mutations that have been reported to date. **(p.Gly77Ser)** reported by Al-Hassnan et al. as the first mutation in ISCA2 (Grey Box). (p.Phe99Leufs*18/p.Ser112Gly) reported by Toldo et al. as the second mutation (Black Boxes). **(p.Ala119Thr)** found by our investigation in an Iranian family (White Box). **b** Secondary structure prediction and comparison of Wild-Type and Mutant ISCA2 protein and presentation of Pfam domain which is involved in Fe-S clusters biosynthesis. In-silico prediction shows that the **(p.Ala119Thr)** mutation eliminates Helix motif in this area

Based on the location of mutations, these three reports neither include nor exclude the possibility of a hotspot for mutations in this gene. However, Multiple Sequence Alignment by ConSurf, indicates that *ISCA2* C-Terminal residues are highly conserved, and also [Fe-S] clusters synthesis domain is coded by C-Terminal. Therefore, more reports of mutations affecting this region of *ISCA2* in the future can be expected (Fig. 4a, b). Additionally, prediction of the Wild-Type and Mutant ISCA2 protein secondary structures showed that the c.355G > A mutation eliminates a helix motif in the Fe-S biosynthesis domain and therefore could strongly affect on its function (Fig. 4b).

Based on clinical observations and neuroradiological findings, genotype-phenotype correlation of *ISCA2* deficiency is clinically overlapped with a wide range of other diseases such as Leukoencephalopathies with Brain Stem and Spinal Cord involvement and Lactate elevation, infantile Metachromatic Leukodystrophy (MLD) and MLD caused by Saposin B deficiency, Krabbe disease, infantile Vanishing White Matter disease, Canavan disease, and Alexander disease [33, 34]. Accordingly, efforts have been made to enable differential diagnosis, but no diagnosis is more accurate and faster than the genetic test.

As stated, impairment in the *ISCA2* function disrupts crucial life pathways, so there is no definitive treatment available so far, other than supportive cares, and early demise occurs, usually.

Overall, we have discovered an Iranian family with the diagnosis of MMDS4 as a result of c.355G > A variant in *ISCA2* gene. Since neurological disease lesions bring lots of psychological issues to the families and also an economic burden to the health system of each country, our report and the two previously mutation reports in *ISCA2* gene suggest to be place this gene in targeted sequencing panels for Prenatal Testing and Preimplantation Genetic Diagnosis in families who are at the risk of infantile leukoencephalopathies.

## Additional file

Additional file 1: **Table S1.** Whole Exome Sequencing statistical analysis. **Table S2.** Reminder heterozygous variants after exclusion phenotype unrelated variants. **Table S3.** Reminder homozygous variants after exclusion phenotype unrelated variants. (DOCX 32 kb)

## Data Availability

The datasets generated and/or analysed during the current study are not publicly available but are available from the corresponding author on reasonable request.

## Abbreviations

GATK: Genome Analysis Toolkit
ISCs: Iron-Sulfur Clusters
MLD: Metachromatic Leukodystrophy
MMDS: Multiple Mitochondrial Dysfunctions Syndrome
MRC: Mitochondrial Respiratory Chain
NAA: N-Acetyl Aspartate
WES: Whole-Exome Sequencing
