# Supplementary material for: A novel ISCA2 variant responsible for an early-onset neurodegenerative mitochondrial disorder: a case report of multiple mitochondrial dysfunctions syndrome 4
Source: BMC Neurol. 2019 Jul 6;19:153. doi: 10.1186/s12883-019-1387-2 (PMC6612116; doi:10.1186/s12883-019-1387-2)
Supplement: Supplementary file 1 — Table S1. Whole Exome Sequencing statistical analysis. Table S2. Reminder heterozygous variants after exclusion phenotype unrelated variants. Table S3. Reminder homozygous variants after exclusion phenotype unrelated variants. (DOCX 32 kb) [file 12883_2019_1387_MOESM1_ESM.docx]

Additional file 1

**Table S1.** Whole Exome Sequencing statistical analysis

|  | Average  Coverage(X) | % Target bp Covered | | | | | |
| --- | --- | --- | --- | --- | --- | --- | --- |
|  |  | **0X** | **≥1X** | **≥5X** | **≥10X** | **≥20X** | **≥100X** |
| Index | 175.571 | 0.159388 | 99.8406 | 99.2349 | 98.2539 | 95.9273 | 86.6968 |

**Table S2.** Reminder heterozygous variants after exclusion phenotype unrelated variants

| **Genomic location** | **Gene** | **Type** | **Protein Consequence** | **Gene specific inheritance_mode** | **Clinical Significance^*^** | **Effect prediction^**^** |
| --- | --- | --- | --- | --- | --- | --- |
| **Chr1:145508244 C/G** | **RBM8A** | **Substitution** | **NM_005105: p.D55E** | **Autosomal Recessive** | **Not Present** | **Possibly Deleterious** |
| **Chr2:170062698 C/T** | **LRP2** | **Substitution** | **NM_004525: p.G2464D** | **Autosomal Recessive** | **Not Present** | **Neutral** |
| **Chr2:219135284 C/A** | **PNKD** | **Substitution** | **NM_015488: p.A9E** | **Autosomal Dominant** | **Not Present** | **Neutral** |
| **Chr2:** **179403709 G/T** | **TTN** | **Substitution** | **NM_001267550: c.C98953A: p.P32985T** | **Autosomal Recessive & Autosomal Dominant** | **Not Present** | **Neutral** |
| **Chr3: 55504577 G/T** | **WNT5A** | **Substitution** | **NM_003392: c.C686A: p.T229K** | **Autosomal Dominant** | **Not Present** | **Neutral** |
| **Chr6:24520643 C/A** | **ALDH5A1** | **Substitution** | **NM_170740: p.H308Q** | **Autosomal Recessive** | **Not Present** | **Neutral** |
| **Chr13:52524238 C/T** | **ATP7B** | **Substitution** | **NM_001243182: p.A768T** | **Autosomal Recessive** | **Not Present** | **Neutral** |
| **Chr15:89868873 G/A** | **POLG** | **Substitution** | **NM_002693: p.T586I** | **Autosomal Recessive** | **Not Present** | **Neutral** |
| **Chr19:55657836 T/G** | **TNNT1** | **Substitution**  **Splicing** | **NM_003283: c.74-2A>C** | **Autosomal Recessive** | **Not Present** | **Most Possibly affecting splicing** |
| **Chr19:17448474 G/T** | **GTPBP3** | **Substitution**  **Splicing** | **NM_032620: c.53+1G>T** | **Autosomal Recessive** | **Not Present** | **Probably no impact on splicing** |
| **Chr1:6529206 TCT/-** | **PLEKHG5** | **Indel**  **Non-frameshift** | [**NM_020631**](https://genome.ucsc.edu/cgi-bin/hgc?hgsid=717809541_8w2okqDrymZxsSPMyV7LhCJ6mIey&g=refGene&i=NM_020631&c=chr1&o=6526151&l=6526151&r=6531643&db=hg19)**: p.E800del** | **Autosomal Recessive** | **Likely Benign** | **Possibly Deleterious** |
| **Chr2:71801349**  **-/CGGAGG** | **DYSF** | **Indel**  **Non-frameshift** | **NM_001130982: c.3292insCGGAGG: p.E1097_G1098insAE** | **Autosomal Recessive** | **Likely Benign** | **Possibly Deleterious** |
| **Chr2:179315738 CG/-** | **PRKRA** | **Indel**  **Frameshift** | [**NM_003690**](https://genome.ucsc.edu/cgi-bin/hgc?hgsid=717809541_8w2okqDrymZxsSPMyV7LhCJ6mIey&g=refGene&i=NM_003690&c=chr2&o=179296140&l=179296140&r=179315958&db=hg19) **: c.19_20delCG: p.A8Rfs*21** | **Autosomal Recessive** | **Likely Benign** | **Deleterious** |
| **Chr2:211421454 -/TTC** | **CPS1** | **Indel**  **Non-frameshift** | **NM_001122633:**  **c.13_15ATCTTC: p.I5_K6insF** | **Autosomal Recessive** | **Not Present** | **Possibly Deleterious** |
| **Chr2:** **241696841 TCC/-** | **KIF1A** | **Indel**  **Non-frameshift** | [**NM_001244008**](https://genome.ucsc.edu/cgi-bin/hgc?hgsid=717809541_8w2okqDrymZxsSPMyV7LhCJ6mIey&g=refGene&i=NM_001244008&c=chr2&o=241653180&l=241653180&r=241759725&db=hg19)**:**  **p.E917del** | **Autosomal Recessive & Autosomal Dominant** | **Benign** | **Possibly Deleterious** |

*Clinical significances were imported from ClinVar.

**An overall interpretation of variants effect on protein functions predicted by SIFT, POLYPHEN2, Mutation Taster, InterVar, Human Splicing Finder and…

**Continue of Table S2**

| **Genomic location** | **Gene** | **Type** | **Protein Consequence** | **Gene specific inheritance_mode** | **Clinical Significance^*^** | **Effect prediction^**^** |
| --- | --- | --- | --- | --- | --- | --- |
| **Chr3:12660096 GC/AT** | **RAF1** | **Indel**  **Non-frameshift** | **NM_002880: c.124_125AT:p.A42I** | **Autosomal Dominant** | **Uncertain Significant** | **Neutral** |
| **Chr3:49395688 CGCCGC/-** | **GPX1** | **Indel**  **Non-frameshift** | **NM_001329455: p.A12_A13del** | **Autosomal Recessive** | **Not Present** | **Possibly Deleterious** |
| **Chr8:96047806 -/A** | **NDUFAF6** | **Indel**  **Splicing** | **NM_152416: c.420+20>A** | **Autosomal Recessive** | **Pathogenic** | **Probably no impact on splicing** |
| **Chr3:58416535 CCCT/TCCC** | **PDHB** | **Indel**  **Non-frameshift** | **NM_000925: c.435_438GGGA** | **Autosomal Recessive** | **Benign** | **Neutral** |
| **Chr4:3076661**  **-/AGC** | **HTT** | **Indel**  **Non-frameshift** | **NM_002111: c.109insAGC:** **p.Q38_P39insQ** | **Autosomal Dominant** | **Not Present** | **Neutral** |
| **Chr4:190876192 AATC/CATT** | **FRG1** | **Indel**  **Non-frameshift** | **NM_004477: c.318_321CATT: p.R106S** | **Autosomal Dominant** | **Not Present** | **Neutral** |
| **Chr16: 1254429 C/A** | **CACNA1H** | **Substitution** | **NM_021098: c.C2422A:p.L808M** | **Autosomal Dominant** | **Not Present** | **Neutral** |
| **Chr17:1631341 GAG/-** | **WDR81** | **Indel**  **Non-frameshift** | **NM_001163809: p.E1033del** | **Autosomal Recessive** | **Not Present** | **Possibly Deleterious** |
| **Chr18:55226380 CATG/TATC** | **FECH** | **Indel**  **Non-frameshift** | **NM_001012515: c.816_819GATA** | **Autosomal Recessive** | **Not Present** | **Possibly Deleterious** |
| **Chr18:77475187 -/GGA** | **CTDP1** | **Indel**  **Non-frameshift** | **NM_048368: p.E582_D583insE** | **Autosomal Recessive** | **Benign** | **Possibly Deleterious** |
| **Chr19:4817288 -/AGG** | **TICAM1** | **Indel**  **Non-frameshift** | **NM_182919: c.1102insCCT: p.P367_S368insP** | **Autosomal Recessive & Autosomal Dominant** | **Benign** | **Possibly Deleterious** |
| **Chr19:13318707 TGCTGC/-** | **CACNA1A** | **Indel**  **Non-frameshift** | **NM_001127222:**  **p.Q2324_Q2325del** | **Autosomal Dominant** | **Not Present** | **Neutral** |
| **Chr19:50762417 CA/TG** | **MYH14** | **Indel**  **Non-frameshift** | **NM_001145809: c.2249_2250TG** | **Autosomal Dominant** | **Benign** | **Possibly Deleterious** |

*Clinical significances were imported from ClinVar.

**An overall interpretation of variants effect on protein functions predicted by SIFT, POLYPHEN2, Mutation Taster, InterVar, Human Splicing Finder and…

**Table S2**

| **Genomic location** | **Gene** | **Type** | **Protein Consequence** | **Gene specific inheritance_mode** | **Clinical Significance^*^** | **Effect prediction^**^** |
| --- | --- | --- | --- | --- | --- | --- |
| **ChrX:153006137 GTCGT/ATCGA** | **ABCD1** | **Indel**  **Non-frameshift** | **NM_000033: c.1744_1748ATCGA** | **X-Linked Recessive** | **Not Present** | **Neutral** |
| **Chr22:** **29191709 C/A** | **XBP1** | **Substitution** | **NM_005080: c.G611T: p.C204F** | **Not Present** | **Not Present** | **Neutral** |
| **ChrX: 47433940 C/A** | **SYN1** | **Substitution** | **NM_133499: c.G1443T: p.L481F** | **X-Linked Recessive &**  **X-Linked Dominant** | **Not Present** | **Neutral** |
| **ChrX: 49113989 C/A** | **FOXP3** | **Substitution** | **NM_014009: c.G349T: p.V117L** | **X-Linked Recessive** | **Not Present** | **Neutral** |
| **Chr2:73613031**  **-/GGA, GGAGGA** | **ALMS1** | **Indel**  **(Compound Heterozygous)** | **NM_015120:**  **c.35insGGAGGA**  **c.35insGGA** | **Autosomal Recessive** | **Uncertain Significant** | **Neutral** |
| **Chr2:166810194**  **-/** **CCGCTCAC,** **CCGCTCAC…** | **TTC21B** | **Indel**  **(Compound Heterozygous)**  **Splicing** | **NM_024753: c.21+10>GTGAGCGGGTGAGCGG**  **c.21+10>GTGAGCGG** | **Autosomal Recessive & Autosomal Dominant** | **Not Present** | **Probably no impact on splicing** |

*Clinical significances were imported from ClinVar.

**An overall interpretation of variants effect on protein functions predicted by SIFT, POLYPHEN2, Mutation Taster, InterVar, Human Splicing Finder and…

**Table S3.** Reminder homozygous variants after exclusion phenotype unrelated variants

| **Genomic location** | **Gene** | **Type** | **Protein Consequence** | **Clinical Significance^*^** | **Effect prediction^**^** |
| --- | --- | --- | --- | --- | --- |
| **Chr14:74961593 G/A** | **ISCA2** | **Substitution** | **NM_194279: p.A119T** | **Not Present** | **Deleterious** |
| **Chr2:73675227 -/CTC** | **ALMS1** | **Indel**  **Frameshift** | **NM_015120: c.1570insCTC: p.S524_L525insP** | **Benign** | **Possibly Deleterious** |
| **Chr2:26477129 -/CTA** | **HADHB** | **Indel**  **Frameshift** | **NM_001281512: c.7insCTA** | **Benign** | **Possibly Deleterious** |
| **Chr8:145738767 C/-^1^** | **RECQL4** | **Indel**  **Non-frameshift** | [**NM_004260.3**](https://www.ncbi.nlm.nih.gov/entrez/query.fcgi?cmd=Search&db=Nucleotide&term=NM_004260.3&doptcmdl=GenBank&tool=genome.ucsc.edu)**: c.2296delC** | **Not Present** | **Deleterious** |
| **Chr11:118898435 C/-^1^** | **SLC37A4** | **Indel**  **Non-frameshift** | [**NM_001164280.1**](https://www.ncbi.nlm.nih.gov/entrez/query.fcgi?cmd=Search&db=Nucleotide&term=NM_001164280.1&doptcmdl=GenBank&tool=genome.ucsc.edu)**: c.527delG: p.L102L** | **Not Present** | **Deleterious** |
| **Chr12:42854205**  **AGAA/GGAG** | **PRICKLE1** | **Indel**  **Non-frameshift** | **NM_153026: c.1899_1902CTCC: p.K632K** | **Not Present** | **Neutral** |
| **Chr13:111154058 TGGT/AGGC** | **COL4A2** | **Indel**  **Non-frameshift** | **NM_001846: c.3804_3807AGGC** | **Not Present** | **Neutral** |
| **Chr14:53619480**  **-/CGCCGC** | **DDHD1** | **Indel**  **Frameshift** | **NM_030637: c.323insGCGGCG** | **Benign** | **Possibly Deleterious** |
| **Chr14:92537354**  **-/CTGCT…** | **ATXN3** | **Indel**  **Frameshift** | **NM_004993: c.916insCAGCAGCAGCAGCAGCAGCAGCAGCAG** | **Benign** | **Possibly Deleterious** |
| **Chr16:1261219 ATCA/GTCG** | **CACNA1H** | **Indel**  **Non-frameshift** | **NM_021098: c.4275_4278GTCG** | **Not Present** | **Neutral** |
| **Chr18:42456670**  **-/TCTT** | **SETBP1** | **Indel**  **Non-frameshift** | **NM_001130110: c.681insTCTT** | **Benign** | **Possibly Deleterious** |
| **Chr2:71062833**  **GCT/GCCT** | **CD207** | **Indel**  **Splicing** | **NM_015717: c.73+10>G** | **Not Present** | **Probably no impact on splicing** |

*Clinical significances were imported from ClinVar.

**An overall interpretation of variants effect on protein functions predicted by SIFT, POLYPHEN2, Mutation Taster, InterVar, Human Splicing Finder and…

1- Low confidence genotyped variant and has a dubious annotation.

**Continue of Table S3**

| **Genomic location** | **Gene** | **Type** | **Protein Consequence** | **Clinical Significance^*^** | **Effect prediction^**^** |
| --- | --- | --- | --- | --- | --- |
| **Chr8: 24811064**  **AGGG/AGGGG** | **NEFL** | **Indel**  **Splicing** | **NM_006158: c.1414-1C>0** | **Not Present** | **Probably no impact on splicing** |
| **Chr8: 143746090**  **CA/-** | **JRK** | **Indel**  **Non-frameshift** | **NM_003724: c.1382_1389TTGTGTGG: p.E460E** | **Not Present** | **Neutral** |
| **Chr11: 111742146**  **-/G** | **ALG9** | **Indel**  **Splicing** | **NM_001077690: c.61-1C>0** | **Not Present** | **No splicing motif alteration** |
| **Chr13: 31287980**  **-/AT** | **ALOX5AP** | **Indel**  **Splicing** | **NM_001204406: c.116+20>AT** | **Not Present** | **Probably no impact on splicing** |
| **Chr16: 76311602**  **-/T** | **CNTNAP4** | **Indel**  **Splicing** | [**NM_001322190.1**](https://www.ncbi.nlm.nih.gov/entrez/query.fcgi?cmd=Search&db=Nucleotide&term=NM_001322190.1&doptcmdl=GenBank&tool=genome.ucsc.edu)**: c.42+1insT** | **Not Present** | **No splicing motif alteration** |
| **Chr21: 46924440**  **CCCAGGCCC/-** | **COL18A1** | **Indel**  **Non-frameshift** | **NM_130445: c.2823_2837CGGCCCCCCAGGCCC** | **Not Present** | **Neutral** |
| **ChrX: 25031806**  **CGC/-** | **ARX** | **Indel**  **Non-frameshift** | **NM_139058: p.A115del** | **Not Present** | **Neutral** |
| **ChrX: 66765158**  **-/GCAGCA** | **AR** | **Indel**  **Non-frameshift** | **NM_001348064: p.Q80_E81insQQ** | **Not Present** | **Neutral** |

*Clinical significances were imported from ClinVar.

**An overall interpretation of variants effect on protein functions predicted by SIFT, POLYPHEN2, Mutation Taster, InterVar, Human Splicing Finder and…
